# A novel mutation in γD-crystallin associated with autosomal dominant congenital cataract in a Chinese family

**Published:** 2011-03-26

**Authors:** Li Wang, Xueli Chen, Yi Lu, Jihong Wu, Boqi Yang, Xinghuai Sun

**Affiliations:** Department of Ophthalmology, Eye and ENT hospital of Fudan University, Shanghai, China

## Abstract

**Purpose:**

To identify the pathogenic gene mutation in a Chinese family with autosomal dominant congenital nuclear cataract.

**Methods:**

After obtaining informed consent, detailed ophthalmic examinations were performed and genomic DNAs were obtained from eleven family members in a three-generation Chinese family with five affected. All exons of candidate genes associated with congenital nuclear cataract were amplified by polymerase chain reaction (PCR) and the PCR products were sequenced in both directions. The hydrophobic property of the mutant protein was analyzed with bioinformatics program ProtScale. The structure homology modeling of the mutant protein was based on Swiss-Model Serve, and its structure was displayed and compared with native γD-crystallin (CRYGD) using the RasMol software.

**Results:**

By sequencing the encoding regions of the candidate genes, a novel mutation (c.110G>C) was detected in exon 2 of *CRYGD*, which resulted in the substitution of a highly conserved arginine by proline at codon 36 (p.R36P). The mutation co-segregated with all patients and was absent in 100 normal Chinese controls. Bioinformatics analysis showed an obvious increase of the local hydrophilicity of the R36P mutant γD-crystallin. The homology modeling showed that the structure of the mutant protein was similar with that of native human γD-crystallin.

**Conclusions:**

The study identified a novel mutation (c. 110G>C) in *CRYGD* associated with autosomal dominant congenital cataract in a Chinese family. It expands the mutation spectrum of *CRYGD* in association with congenital cataract.

## Introduction

Congenital cataracts are one of the common eye disorders leading to visual impairment or blindness in children worldwide. Congenital cataract may be inherited or familial, either as an isolated form or as a part of a syndrome, such as Nance-Horan syndrome. In isolated inherited congenital cataract, autosomal dominant (AD), autosomal recessive (AR), and X-linked inheritance have been reported [1,2]. Along with the development of molecular genetics, more than 20 genes have been identified to be involved in isolated cataract formation. Many of them encode crystallins [3-13], such as αA-crystallin (*CRYAA*), αB-crystallin (*CRYAB*), βA1/A3-crystallin (*CRYBA1/A3*), βA4-crystallin (*CRYBA4*), βB1-crystallin (*CRYBB1*), βB2-crystallin(CRYBB2), βB3--crystallin(CRYBB3), γC-crystallin(CRYGC), γD-crystallin (*CRYGD*), and γS-crystallin (*CRYGS*).

Among these crystallin genes, the number of reported mutations in *CRYGD* and correlative phenotypes in human are extensive, such as R14C mutation associated with progressive juvenile-onset punctate cataracts, nuclear cataract and coralliform cataract [12,14], R58H mutation associated with aculeiform cataract and coral-like cataract [11,15], R36S associated with nuclear cataract and crystal cataract [16,17], P23T associated with lamellar cataract, cerulean cataract, coral-like cataract, a flaky, silica-like nuclear cataract, fasciculiform cataract and coralliform cataract [18-23], W156X associated with central nuclear cataract [20], P23S associated with polymorphic congenital cataract [24], G61C causing autosomal dominant congenital coralliform cataracts [25], Y56X associated with nuclear cataract [26], R77S associated with a juvenile autosomal dominant anterior polar coronary cataract [27], E107A associated with nuclear congenital cataract [28], R15S co-segregated with coralliform cataract [29], G165fs associated with nuclear cataract [30], Y134X associated with microcornea-cataract [31], R140X related with inherited pediatric cataract [32]. In mice, mutations in *Crygd* have been identified and shown to lead to dominant, congenital cataracts [33]: *Crygd^Lop12^*, *Crygd^Aey4^*, *Crygd^ENU4011^*, *Crygd^ENU910^*, and *Crygd^K10^*.

In this study, we reported a novel mutation in CRYGD **(**p.R36P) which is related with congenital cataract in a Chinese family.

## Methods

### Clinical evaluation and DNA specimens

**Figure 1:**
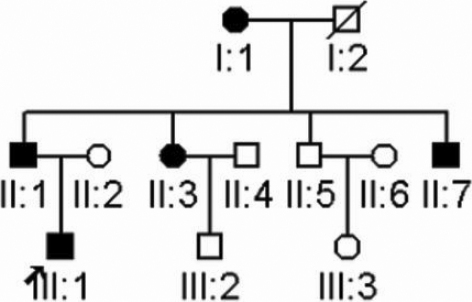
Pedigree of inherited nuclear cataract. Squares and circles symbolize males and females, respectively. Clear and blackened symbols denote unaffected and affected individuals, respectively. The arrow indicates the proband.

A three-generation family with autosomal dominant congenital nuclear cataract was ascertained (Figure 1). After explanation of the nature and possible consequences of the study, eleven individuals participated in the study. The study was performed with informed consent and following all the guidelines for experimental investigations required by the Institutional Review Board of Eye and EENT Hospital of Fudan University. The ophthalmologic examinations, including visual function and dilated slit-lamp examination, were performed by ophthalmologists. Blood samples were collected and leukocyte genomic DNA was extracted.

### Mutation detection

All the exons of candidate genes which were associated with autosomal dominant congenital nuclear cataract, including *CRYAA, CRYBA1/A3, CRYBB2, CRYBB3, CRYGC, CRYGD, CRYGS, GJA3*, and *GJA8,* were amplified by PCR. The primers used are listed in Table 1. The PCR products were sequenced in both directions with an ABI 3130XL Genetic Analyzer (Applied Biosystems, Foster City, CA). The results were analyzed using Chromas (version 2.23) software and compared with the reference sequences in the NCBI gene bank.

**Table 1:** Primers for PCR amplification of exons of candidate genes and the size of the PCR products.

**­­­Gene**	**Exon**	**Primer direction**	**Primer sequence (5′-3′)**	**Fragment size (bp)**	**­­­Gene**	**Exon**	**Primer direction**	**Primer sequence (5′-3′)**	**Fragment size (bp)**
*CRYAA*	1	F	acttgtcccagccacgttt	515		3	F	ttcccggtatgtcctagcag	511
		R	ctctgcaaggggatgaagtg				R	ctggtgtctcaatccccaac	
	2	F	aaccagccacctgaccatag	637		4	F	atcaaccagctttggaggaa	527
		R	acattagctcgggaatggtg				R	cttgcagtgagctgagatcg	
	3	F	caggggcatgaatccataaa	551		5	F	acggtgttgagtgtgaatgg	679
		R	taagctctcctggctgctct				R	ggctctgcctgaaaggatta	
*CRYBA1/A3*	1	F	gggcctctctggatttctgt	404	*CRYGC*	1	F	tttccagtgaatgcaggatg	672
		R	gctagggcagtggttattgc				R	tctgctgtttttgtgcatgtt	
	2	F	gcagaggttgcagtgaagtg	516		2	F	cgcagcaaccacagtaatct	641
		R	caatggcatccacagtcatc				R	caacgtctgaggcttgttca	
	3	F	actctgggcaaatgaacacc	547	*CRYGD*	1	F	gagagaatgcgaccaaaacc	742
		R	tctttatccagcccctgaaa				R	gcttatgtggggagcaaact	
	4	F	cctgtcaactcattcctcaactc	557		2	F	tgtgctcggtaatgaggagtt	586
		R	tgggctcttgagtatccactt				R	cacatcttggttgccatttg	
	5	F	tggttggctgcattttgtta	609	*CRYGS*	1	F	tgtctgggttttggtttcttg	480
		R	gcatgtctgggggagataaa				R	cggtaattctgttaagggtgga	
	6	F	cccccagacatgcctctat	648		2	F	tgcttcatgttcagccttca	662
		R	ttacactccagcctgagcaa				R	ctcaagaggcagagacagca	
*CRYBB2*	1	F	cagaggggagtggtctcaag	540		3	F	gtgctgtctctgcctcttga	852
		R	caaagccagaggctggtact				R	cctctcacagggttgtaagga	
	2	F	agaggagaaatgcaggctca	600	*GJA3*	1	1F	ttgtgtagtgcctgctcgtc	711
		R	gcagacaggagcaagggtag				1R	agctcgaagccgtacagaaa	
	3	F	atggaaattggcaaacgcta	586			2F	aaagagagggaggaggagga	668
		R	tcctggtccccagacctcca				2R	gcccagttctgctcagtcat	
	4	F	tagacacgtagtgggtgcac	405			3F	gctggaagaagctcaagcag	803
		R	cagaggtcagcagagcacac				3R	aagcattgaacacggaaacc	
	5	F	tatcacccccttgctctgac	1105	*GJA8*	1	1F	tctgcacaaaggaagcactg	690
		R	cccctgagagtgactgtgct				1R	cggaacccgtacaggaagta	
*CRYBB3*	1	F	gagcctcagagttcccctct	512			2F	gtgctgcagatcatcttcgt	825
		R	gcagcaaagtcatgaagcaa				2R	tgcttcctccttcttctctcc	
	2	F	tgaagttcctgaaggcgttt	501			3F	tgagaaatccctccactcca	752
		R	aggtatcctgggattttctgc				3R	gtagcccttatgctggatgc	

The letters F and R indicate forward and reverse primers, respectively.

### Bioinformatics analysis

The wild-type and mutant CRYGD protein sequences were analyzed with PolyPhen to predict whether the amino acid substitution affects the structure and function of proteins, with a position-specific independent counts (PSIC) score difference for two amino acid variants. The hydrophobic properties of mutant and wild-type CRYGD were analyzed with ProtScale. The structure homology modeling of the mutant protein was modeled by Swiss-Model Serve [34], and its structure was displayed and compared with native human CRYGD using RasMol software. The structure of native human CRYGD (1hk0) was obtained from the PDB database.

## Results

### Clinical evaluations

**Figure 2:**
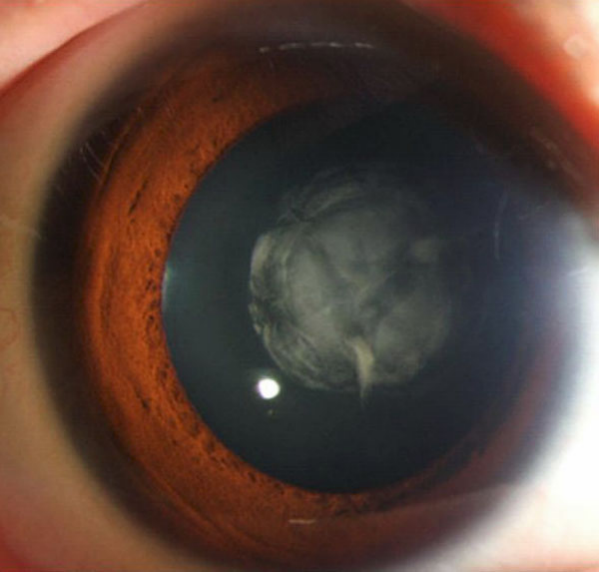
Slit-lamp photograph of the proband. This photograph showed a cataract characterized as a central nuclear opacity of the lens.

There were five patients in this three-generation family (Figure 1). Cataract was characterized as bilateral, white, central nuclear opacities (Figure 2) in the affected members. There were no other ocular or systemic abnormalities. The affected individuals I1, II1, and III1 have had cataract surgery. An autosomal dominant inheritance mode of the cataract was supported by the presence of affected individuals in each of the three generations, and male-to-male transmission.

### Mutation detection

**Figure 3:**
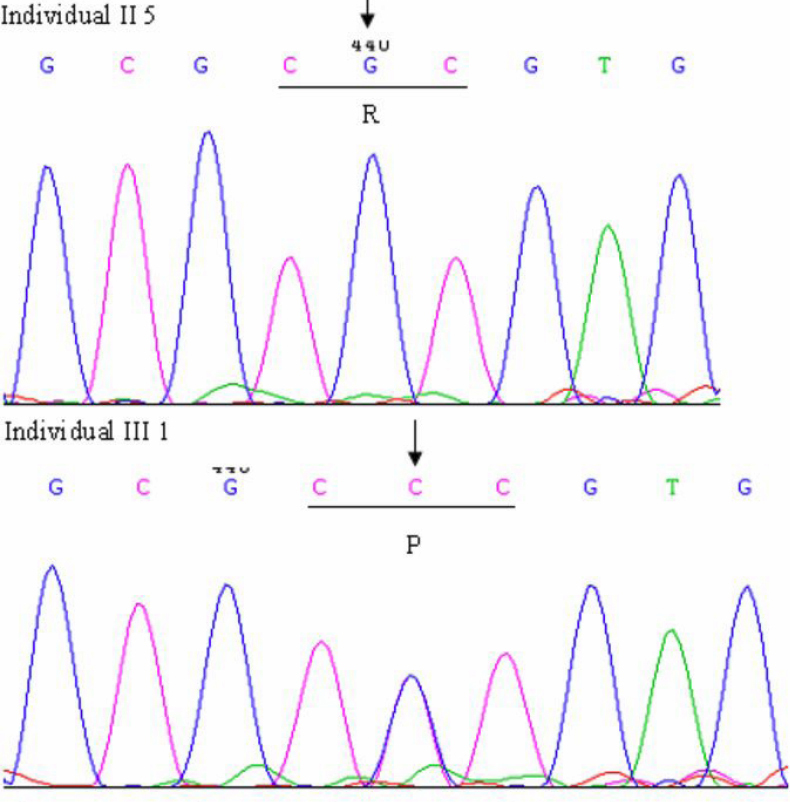
Forward sequence chromatogram of exon 2 of *CRYGD*. The arrow indicates the G >C transition. Individual II5 is unaffected (upper panel), III1 is affected (lower panel). The encoded amino acid at codon 36 (underlined) is indicated, CGC encodes arginine (R), CCC encodes proline (P).

By bidirectional sequencing of amplified exons of the candidate genes, we found a heterozygous missense mutation, G>C at position 110 in exon 2 of *CRYGD* (NM_006891) in affected individuals, but not in unaffected individuals. This change led to the substitution of arginine by proline at position 36 (p.R36P; Figure 3). This mutation was not found in 100 unrelated control individuals. No other sequence variant was found.

### Bioinformatics analysis

**Figure 4:**
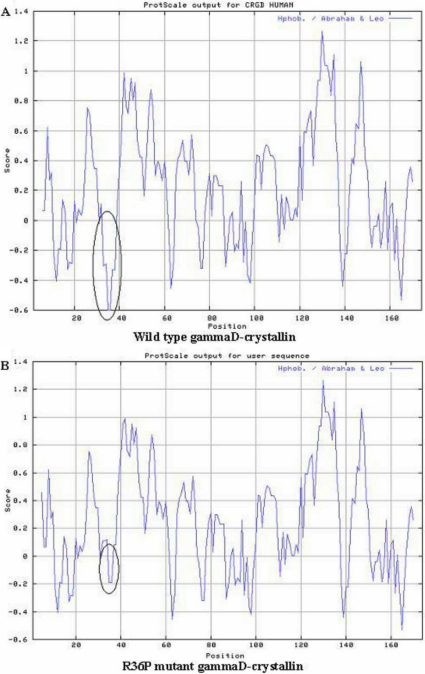
Hydrophilicity analysis of the wild-type CRYGD (**A**) and mutant CRYGD (**B**). The 36th amino acid and its neighboring locations are marked by the circle, there is an obvious increase in the local hydrophilicity of the R36P mutant CRYGD.

**Figure 5:**
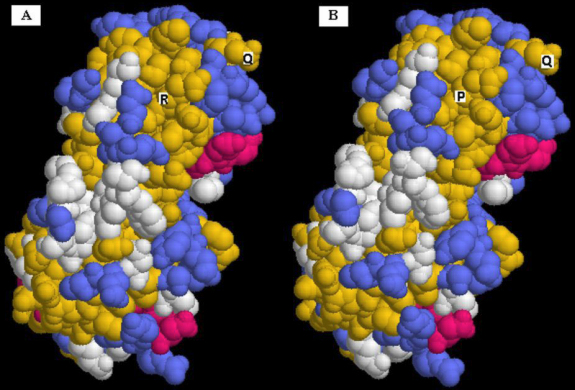
Structure homology modeling and comparison of mutant protein and native human CRYGD (1hk0). **A**: Native human CRYGD and **B**: Mutant protein. Red, yellow, and blue indicate α-helix, β-sheet, and β-turn, respectively, white indicates other residues. R, P and Q represent Arg36, Pro36, and Gln12, respectively.

PolyPhen analysis showed that the substitution in CRYGD (p.R36P) had a PSIC score difference of 2.796, which meant that “this variant is predicted to be probably damaging.” It is with high confidence supposed to affect protein function or structure. The change in hydrophobicity of the mutant and wide protein is shown in Figure 4. An obvious increase can be seen in the local hydrophilicity of R36P mutant CRYGD. The homology modeling showed that the second structure of the mutant protein was similar with that of native human CRYGD (Figure 5).

## Discussion

In a Chinese family with congenital nuclear cataract, we identified a novel mutation c.110G>C in *CRYGD*, leading to the substitution of arginine by proline (p. R36P). This mutation co-segregated with the phenotype and was not found in 100 unrelated control individuals.

*CRYGD* is one of only two gamma-crystallin genes to be expressed at high concentrations in the human lens. *CRYGD* which encodes a 174-amino acid protein is located on chromosome 2q33.3. CRYGD is an important structural protein, its high concentration and conserved conformational symmetry are associated with high refractive index of the lens, which keeps the lens transparent.

Most of the mutations of *CRYGD* which were reported in different ancestral families with congenital cataract actually involve an arginine residue in conserved positions, such as R14C, R15S, R58H, R36S, R77S, and R140X. The R36S mutation of the processed, initiation-methioine-lacking protein was first described in a Czech 5-year-old boy with crystal cataract caused by deposition of crystallized protein [16]. The same mutation was detected in a Chinese family with nuclear golden crystal cataract [17]. The X-ray structure of CRYGD revealed that the protein fold of the p.R36S mutant protein was almost identical to that of bovine CRYGD, but this mutation changed the solvent-accessible surface characteristic, decreased the charge and increased the local hydrophobicity. Protein crystallography study displayed the normal crystals cannot form with wild-type protein because of steric hindrances imposed by the bulky Arg36 side chains [16]. Pande et al. [35] showed the p.R36S mutation dramatically lowered the solubility of the protein, the mutant protein were more prone to crystallization than wild-type human CRYGD protein. The P23T, P23S, and R58H mutant protein was also found to be less soluble than wild type human CRYGD [35,36].

In our study, we detected a novel mutation in the same codon (p.R36P) to be the basis of congenital nuclear cataract without crystal manifestation in a Chinese family. The different mutation of the same condon was also reported in the *CRYGD* gene, such as P23T and P23S, which were related with different cataract [18-24]. For the different clinical manifestations, it is presumed that modifying factors or epistatic elements, such as the difference in the gene promoter site, might regulate *CRYGD* expression in the lens.

The residue 36 arginine is highly conserved, a highly polar and hydrophilic residue.In the p.R36P CRYGD mutant, it was replaced by less polar, hydrophobic residues proline. The prediction by ProtScale analysis at Expasy showed an obvious increase of local hydrophobicity around the site of R36P mutation. The homology modeling showed that the second structure of the mutant protein was similar with that of native human CRYGD. It can be presumed that R36P mutation would result in incorrect solvent-accessible surface characteristics and lower the solubility of the protein in the affected individuals, like R36S and other dominantly inherited mutations reported in CRYGD. The activity of R36P mutation identified in our study to the CRYGD needs to be further certificated.

In conclusion, we identified a novel mutation (R36P) in CRYGD associated with autosomal dominant nuclear cataract in a Chinese family. This finding expands the mutation spectrum of *CRYGD* in association with congenital cataract.
